# Nitrogen‐Driven Changes in Canopy Structure Enhance Productivity and Efficiency of Guineagrass ( *Megathyrsus maximus* ) cv. BRS Quênia Across Contrasting Growing Seasons

**DOI:** 10.1002/pei3.70212

**Published:** 2026-10-23

**Authors:** Marislayne de Gusmão Pereira, Gelson dos Santos Difante, Denise Baptaglin Montagner, Valéria Pacheco Batista Euclides, Alexandre Romeiro de Araújo, Jéssica Gomes Rodrigues, Gabriela Oliveira de Aquino Monteiro, Juliana Caroline Santos Santana, Amanda Eunice Silva Moura, Luís Carlos Vinhas Ítavo

**Affiliations:** ^1^ Faculty of Veterinary Medicine and Animal Science Federal University of Mato Grosso do Sul Campo Grande Mato Grosso do Sul Brazil; ^2^ Brazilian Agricultural Research Corporation Embrapa Beef Cattle Campo Grande Mato Grosso do Sul Brazil; ^3^ Department of Animal Science São Paulo State University “Júlio de Mesquita Filho”–UNESP Jaboticabal São Paulo Brazil; ^4^ Graduate Program in Animal Production Federal University of Rio Grande do Norte Macaíba Rio Grande of Norte Brazil

**Keywords:** agronomic efficiency, *Megathyrsus maximus*, nutritive value, sustainable intensification

## Abstract

Understanding the responses of forage cultivars to nitrogen (N) fertilization is essential for developing efficient strategies for the sustainable intensification of pasture‐based livestock systems. This study aimed to evaluate the effects of increasing N doses on forage production, canopy structure, nutritive value, and agronomic efficiency of *Megathyrsus maximus* cv. BRS Quênia pastures under intermittent stocking. The experiment was conducted over 2 years using a randomized complete block design with four N doses (75, 150, 225, and 300 kg N ha^−1^ year^−1^) and four replications. The following variables were assessed: grazing interval, canopy height, tiller population density (TPD), forage mass (FM) and accumulation (FA), morphological composition, nutritive value, and agronomic efficiency index (AEI). Increasing N doses reduced the grazing interval from 60 to 25 days and increased the FAR from 71 to 169 kg DM ha^−1^ day^−1^. Forage production (FP) increased linearly, reaching 21.060 kg ha^−1^ year^−1^ of dry matter at the highest N doses in Year 2, representing an increase of up to 48.5% compared to Year 1. The AEI was higher in the second year, with a maximum value of 486% observed at 300 kg N ha^−1^. Higher N doses increased TPD (565 tillers m^−2^), with greater leaf proportion (49%) and lower dead material (16%). In contrast, the lowest N dose resulted in reductions of up to 40% in TPD and 48% in FP in Year 2. Nutritive value was also influenced by N doses, with increases in crude protein (up to 14.7%) and digestibility (up to 59.6%). The lowest N dose (75 kg N ha^−1^ year^−1^) compromised productivity in Year 2, indicating that at least 150 kg N ha^−1^ year^−1^ is required to maintain pasture stability. Above 225 kg N ha^−1^ year^−1^, agronomic benefits were marginal relative to economic costs and environmental risks. Therefore, the range of 150–225 kg N ha^−1^ year^−1^ represents the optimal balance between productive intensification, economic viability, and environmental sustainability under climatic variability.

## 1 Introduction

The productivity of livestock production systems based on forage, the main feed source for ruminants, can be enhanced through the adoption of improved technologies and management practices (Wani and Habib 2022). Currently, animal science faces the challenge of increasing production while optimizing the use of resources and land (Röös et al. 2017), reducing environmental impacts (Govindasamy et al. 2023; Komainda et al. 2024; Nyameasem et al. 2024). In this context, pasture intensification has been identified as a key strategy to improve land use efficiency and increase forage production in tropical regions (Cardoso et al. 2020).

National and international research institutions have focused on developing improved forage cultivars and promoting their adoption, aiming to increase herbage production and enhance livestock productivity (Mwendia et al. 2022). However, the successful adoption of these new cultivars depends on the development of appropriate management strategies, including recommendations for nitrogen fertilization and grazing management. In Brazil, the breeding program for *Panicum maximum* (syn. *Megathyrsus maximus* ) released the cultivar BRS Quênia (“Quênia guineagrass”) in 2017, an interspecific hybrid developed to diversify pastures, intensify animal production, and meet the demand for cultivars with high forage yield, good nutritive value, and intermediate growth habit (Jank et al. 2017).

In addition to improved cultivars, nitrogen (N) fertilization is a widely adopted strategy for intensification. *Panicum maximum* is characterized by high productivity and nutritive value, making it suitable for intensified forage systems; however, it has high soil fertility requirements, particularly for nitrogen (Paciullo et al. 2017). Previous studies have shown that N fertilization increases herbage production and quality in tropical regions (Escarela et al. 2017; Galindo et al. 2019; Euclides et al. 2022), significantly affects production costs (Sales et al. 2020), and, when mismanaged, may lead to relevant environmental impacts (Galindo et al. 2022). Therefore, understanding the responses of new cultivars to N fertilization is essential for developing efficient nitrogen use strategies.

The integration of nitrogen fertilization with sward structure control has been recommended to improve grazing efficiency and pasture utilization (Gomide et al. 2019; Euclides et al. 2022). Managing pastures based on light interception allows tropical grasses to reach maximum herbage accumulation by interrupting regrowth at the optimal stage (Pedreira et al. 2017). Several studies have identified 95% light interception (LI) as the maximum threshold for interrupting regrowth and initiating grazing in tropical forage grasses (Carnevalli et al. 2006; Barbosa et al. 2007; Da Silva et al. 2015; Alvarenga et al. 2020). However, studies conducted during the development and release of new cultivars often lack detailed information on pasture management based on this concept (Tesk et al. 2020). Although Quênia guineagrass has been evaluated for its agronomic performance, its response to increasing nitrogen fertilization under a grazing management strategy based on the 95% LI criterion has not been systematically investigated. This represents an important knowledge gap for defining efficient management strategies for this cultivar.

Thus, understanding the responses of Quênia guineagrass to different N doses is essential to support strategies that ensure productive balance in livestock systems. This study aimed to quantify the effects of increasing N doses on sward structure, forage accumulation, nutritive value, and agronomic efficiency of Quênia guineagrass. We hypothesized that management based on 95% LI combined with increasing N doses would (i) modify sward structure, (ii) increase forage mass, (iii) enhance forage accumulation, (iv) improve forage nutritive value, and (v) increase agronomic efficiency as N supply increases.

## 2 Materials and Methods

### 2.1 Site, Climate, and Soil

The experiment was conducted at Embrapa Beef Cattle, Campo Grande, MS, Brazil (20°27′ S, 54°37′ W; 530 m a.s.l.), from October 2020 to April 2022. The regional climate is classified as tropical savanna (Aw, Köppen), with a well‐defined rainy season and a dry period from April to September. Precipitation and minimum, mean, and maximum temperatures (Figure 1) were recorded at a meteorological station located approximately 3 km from the experimental area. Soil water balance was estimated (Figure 2) using the Thornthwaite and Mather (1955) method, assuming a water holding capacity of 150 mm.

**FIGURE 1:**
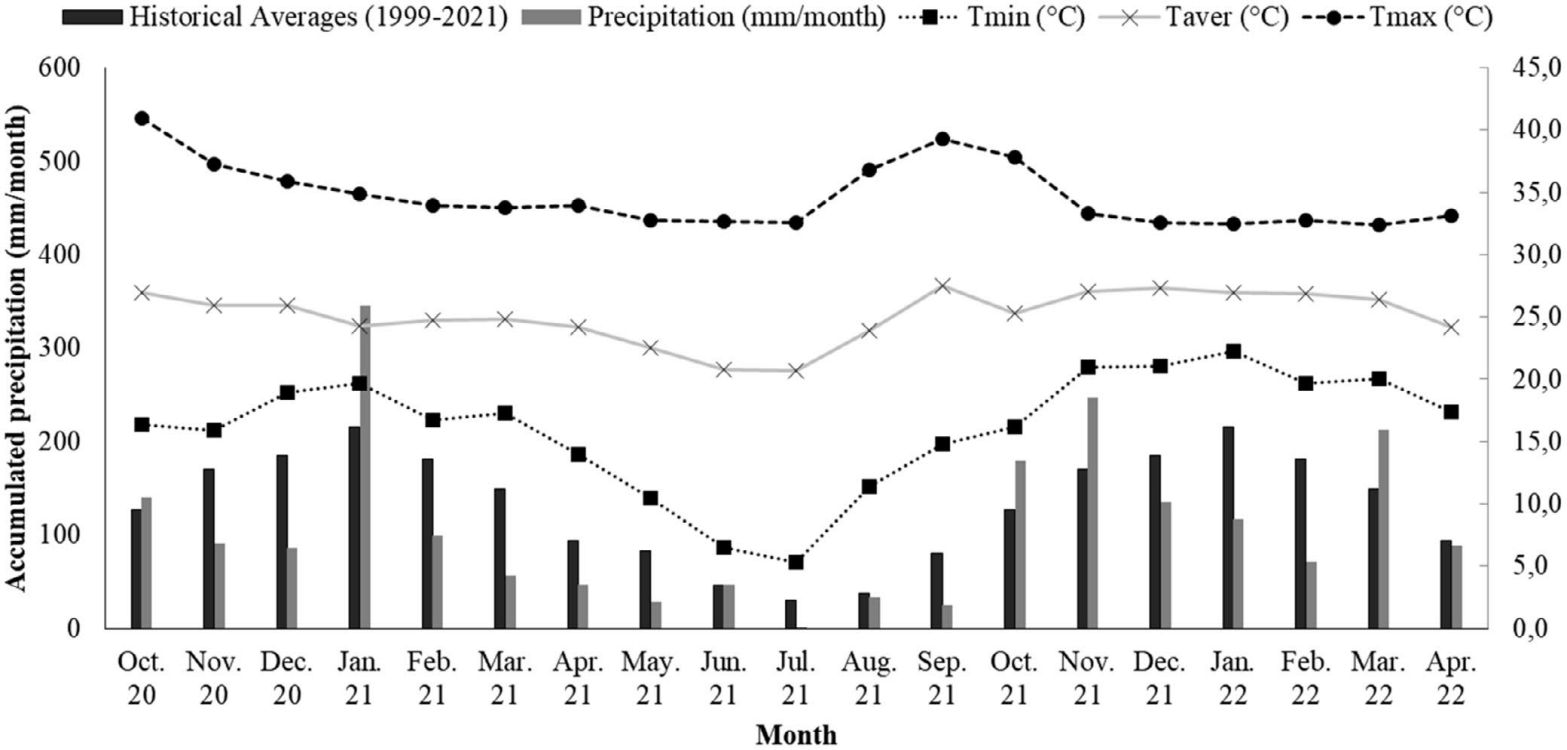
Monthly precipitation and minimum, average, and maximum temperatures during the experimental period and historical average precipitation over 23 years.

**FIGURE 2:**
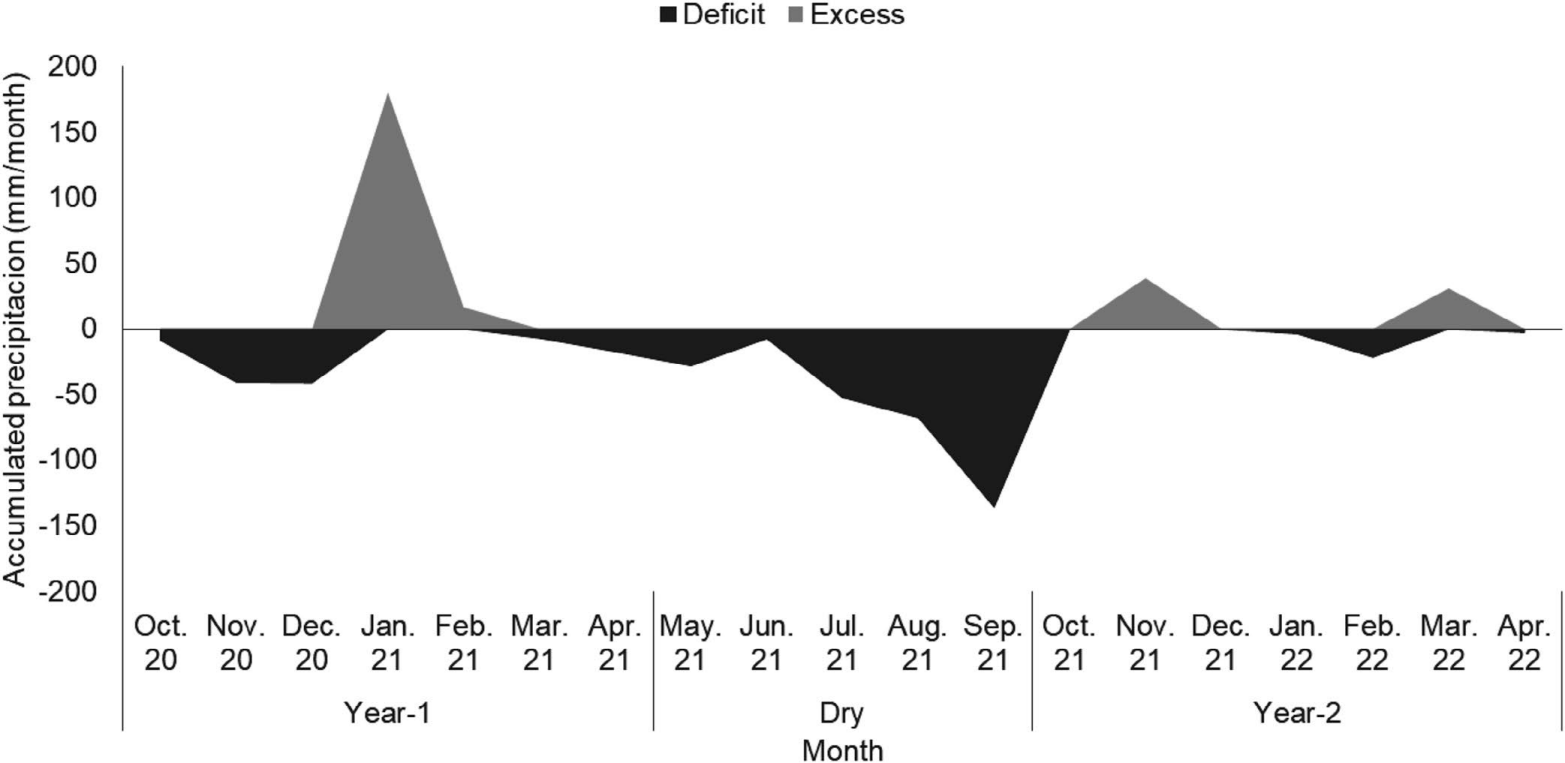
Monthly soil water balance of the experimental area for a soil water storage capacity of 150 mm, from October 2020 to April 2022.

The soil is classified as a dystrophic Red Nitosol with clayey texture, acidic pH, low base saturation, and high aluminum concentration (Santos et al. 2018). Based on soil chemical analysis (Table 1), pastures were fertilized in October 2020 and October 2021 with 60 kg ha^−1^ year^−1^ of P_2_O_5_ and 60 kg ha^−1^ year^−1^ of K_2_O.

**TABLE 1:** Results of soil chemical analysis.

Layers	Ph	P	K^+^	Ca^2+^	Mg^2+^	Al^3+^	H + Al^3+^	S	T	t	MO	V	m
(cm)	(CaCl_2_)	(mg/dm^−3^)	(cmol dm^−3^)								%		
0–10	4.9	6.3	0.4	2.6	1.9	0.3	5.8	4.9	10.7	5.2	4.0	45.1	8.3
0–20	4.9	5.1	0.2	2.5	1.7	0.5	5.3	4.7	9.7	4.9	3.6	44.1	13.1
20–40	4.9	4.5	0.2	2.7	1.8	0.3	5.4	4.8	10.2	5.1	3.9	46.3	7.5

*Note:* S = sum of bases, T = potential CEC, t = effective CEC, MO = organic matter, V = base saturation; m = Al saturation. P and K—Mehlich I; Ca and Mg—Mehlich III; Al—KCl; H + Al—SMP; MO—modified South Dakota.

### 2.2 Experimental Design and Treatments

The study was conducted in a pasture of *Megathyrsus maximus* Quênia guineagrass, established in March 2017 and managed under occasional grazing during 2018, 2019, and early 2020. In September 2020, a uniformity grazing was performed to standardize sward conditions before the experimental period. The experimental area comprised 0.72 ha, divided into four blocks, each subdivided into four paddocks (0.045 ha each), totaling 16 paddocks.

A randomized complete block design was adopted, with four treatments and four replications. Treatments consisted of four nitrogen doses: 75, 150, 225, and 300 kg N ha^−1^ year^−1^ (N75, N150, N225, and N300). Nitrogen fertilization was applied during the rainy season (November to March) using urea as the nitrogen source. Fertilizer applications were divided according to the N dose, with a maximum of 75 kg N ha^−1^ year^−1^ applied per application. Accordingly, the N75, N150, N225, and N300 treatments received one, two, three, and four applications, respectively.

### 2.3 Defoliation Agents

The experimental protocol involving animals was approved by the Ethics Committee on Animal Use (CEUA) of Embrapa Beef Cattle (Protocol no. 003/2018). Twenty Caracu steers (average body weight of 400 kg and approximately 24 months of age) were used as grazing animals. These animals were kept in a reserve area planted with *Megathyrsus maximus* cv. Massai until each Quênia guineagrass paddock reached the predetermined pre‐grazing targets.

Grazing frequency was determined when the sward reached 95% LI during regrowth, and grazing intensity was set at 50% of pre‐grazing height. Intermittent stocking was adopted following the mob grazing approach (Allen et al. 2011).

The instantaneous stocking rate was adjusted considering the 1–2‐day occupancy period and the post‐grazing target height. Animals were allocated to each paddock according to forage availability to achieve the predetermined post‐grazing height (50% of the pre‐grazing height). Canopy height was monitored frequently during the grazing period, and the number of animals was adjusted when necessary to ensure that the target stubble height was reached within the planned grazing duration.

### 2.4 Measurements

#### 2.4.1 Light Interception, Sward Height, and Grazing Interval

LI was monitored using a canopy analyzer (AccuPAR Linear PAR/LAI ceptometer, Model PAR‐80; Decagon Devices) at weekly intervals, increasing to daily measurements as the pre‐grazing target approached. Measurements were taken at 10 random points per paddock, with one reading at ground level and another above the canopy.

Canopy height pre (Pre‐CH) and post‐grazing (Post‐CH) were measured simultaneously at 20 random points per paddock using a graduated ruler, based on the average curvature of the leaves around the ruler. Post‐grazing heights were recorded at the time the animals were removed. Grazing intervals (GI) were calculated as the number of days between animal removal and the moment the pasture again reached 95% LI.

### 2.5 Herbage Production, Nutritive Value, and Agronomic Efficiency

Pre (FMpre) and post‐grazing (FMpos) forage mass was estimated by harvesting three samples from representative areas corresponding to average sward height, using 1 m^2^ quadrats. Samples were cut at ground level using a brush cutter (KA 85R, Stihl). Samples were weighed and divided into two subsamples: one dried at 65°C to constant weight for dry matter (DM) determination. The other portion was separated into leaf blade, stem with sheath, and dead material, and subsequently dried at 55°C to determine the percentage of leaf (LP), stem (SP), and dead material (DMP), as well as the leaf:stem ratio (LSR) and the green:dead ratio (GDR).

Dried leaf and stem samples were ground to pass a 1 mm screen and analyzed by near‐infrared reflectance spectroscopy to determine crude protein, neutral detergent fiber, acid detergent lignin, and in vitro organic matter digestibility. Calibration procedures are described in Campos et al. (2024).

Forage accumulation (FA) was calculated as the difference between FMpre and the previous FMpos, considering only green fractions. Forage accumulation rate (FAR) was obtained by dividing accumulation by the GI. Annual forage production (FP) was calculated as the sum of accumulation across all grazing cycles. Agronomic efficiency index (AEI) was calculated according to Martuscello et al. (2018), using the 75 kg N ha^−1^ treatment as the reference, since an unfertilized control was not included in the experiment. Because unfertilized pastures (0 kg N ha^−1^) are no longer considered a sustainable management option due to soil nutrient mining, the lowest fertilization dose (75 kg N ha^−1^) was adopted as the baseline treatment.

$$\mathrm{AEI}=\frac{{\mathrm{FP}}_{\text{dose}\;n}-{\mathrm{FP}}_{\text{dose}\;75}}{{\mathrm{FP}}_{\text{dose}\;75}}\times 100$$

where AEI: agronomic efficiency index and FP: forage production.

### 2.6 Tiller Density

Tiller population density (TPD) was assessed by counting tillers in an area of 1 m^2^ at three points representative of the average pasture height, per paddock. These areas were chosen to reflect the average condition of the pasture, and counts were conducted whenever the pastures achieved the pre‐grazing condition.

### 2.7 Statistical Analysis

Data were collected for each grazing cycle and grouped into Year 1 (October 2020 to March 2021) and Year 2 (October 2021 to March 2022). Data were analyzed using analysis of variance for a randomized complete block design. The statistical model used was:

Yijk=𝜇+𝐵𝑗+𝐼𝑃𝑖+𝑌𝑘+(IP×𝑌)𝑖𝑘+𝜀𝑖𝑗𝑘

where *Yijk* is the observed value in the plot subjected to *i*—dose of N, in *j*—block and *k*—year; *μ* is the overall mean; *Bj* is the random effect of the block (*j* = 1, 2, 3, 4); IP*i* is the fixed effect of the N doses (*i* = 75, 150, 225, and 300 kg N ha^−1^ year^−1^); *Yk* is the fixed effect of the year (*k* = Year 1 and Year 2); *Yk* is the fixed effect of the interaction between the N doses and the year; (IP × *Y*)*ik* is the fixed effect of the interaction between the N doses and the year; and *εijk* is the residual error term.

The year was considered a fixed effect because the differences between the years and their interaction with N doses were of interest due to the contrasting precipitation patterns (Figure 1). The data were subjected to analysis of variance using the PROC MIXED procedure in SAS OnDemand for Academics, considering N, Year, and their interaction as fixed effects and blocks as random effects.

When a significant interaction between nitrogen dose and year was detected (*p* ≤ 0.05), means were compared using Tukey's test at the 5% probability level. In the absence of interaction, nitrogen effects were evaluated using regression analysis, testing linear, and quadratic models, with model selection based on statistical significance and biological interpretability. Differences between years were assessed using the *F*‐test from the analysis of variance.

The complete dataset used for the statistical analyses is available in Data S1.

## 3 Results

### 3.1 Light Interception, Grazing Interval, and Canopy Height

The 95% LI target was consistently achieved, regardless of the N dose and experimental year, confirming the efficiency of the adopted management criterion. The GI adjusted to the decreasing linear regression equation with a decrease of 0.13 days for each kg N ha^−1^ year^−1^ added, going from 60 days in N75 to 25 days in N300 (*Ŷ* = 61.326588 − 0.139497*x*) (Figure 3). The number of grazing cycles (GC) increased linearly with N fertilization, with an increase of 2.25 grazing cycles for each 75 kg N ha^−1^ year^−1^ increment, rising from four cycles at N75 to 11 cycles at N300 (*Ŷ* = 2.50 + 2.2*x*) (Figure 1).

**FIGURE 3:**
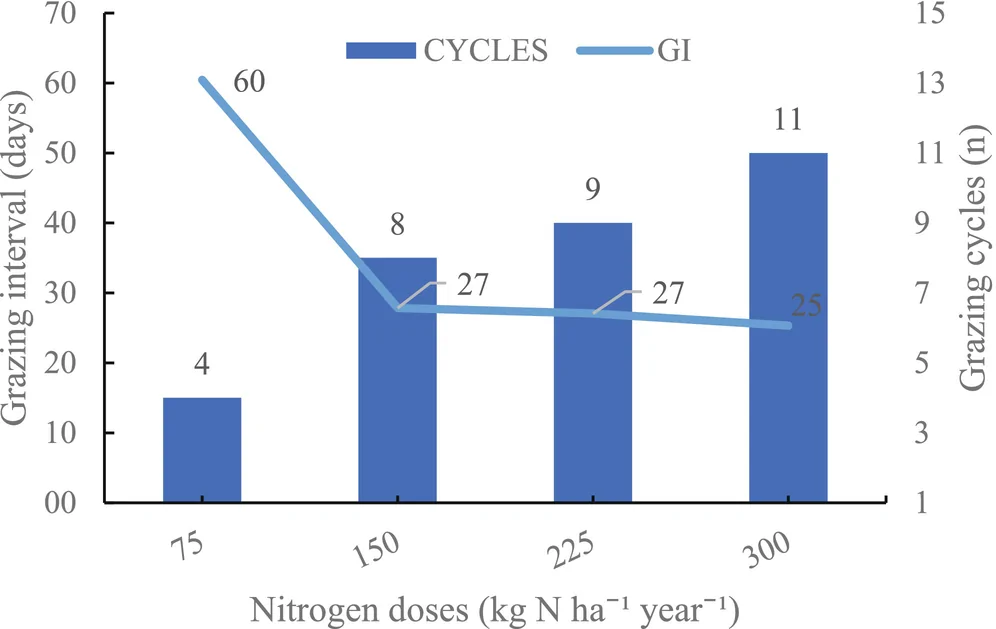
Grazing cycles and intervals for Quênia guineagrass pastures fertilized with increasing doses of nitrogen.

There was an interaction between N dose and year for pre‐CH (*p* < 0.0001) and post‐CH (*p* = 0.0005) conditions, and for TPD (*p* < 0.0001). In Year 2, there was a linear reduction of 3.5 and 2.1 cm in pre‐CH and post‐CH, respectively, with increasing N doses. Comparisons between experimental years within each N fertilization dose revealed increases in pre‐CH and post‐CH for N75, N150, and N225, whereas these variables remained unchanged in N300 (Table 2).

**TABLE 2:** Canopy height pre and post‐grazing and tiller population density of Quênia guineagrass subjected to nitrogen fertilization and evaluated over 2 years.

Year	Nitrogen doses (kg N ha^−1^ year^−1^)				SEM	*p*lin	*p*quad	Equation	*R* ^2^
	75	150	225	300					
Pre‐CH (cm)									
Year 1	59.6b	56.7b	56.7b	56.7a	1.29	ns	ns	*Ŷ* = 57.47	ns
Year 2	72.7a	65.1a	62.1a	58.8a	1.33	0.000	0.109	*Ŷ* = 75.87 − 0.05*x*	0.94
Post‐CH (cm)									
Year 1	29.5b	28.4b	27.7b	28.31a	0.68	ns	ns	*Ŷ* = 28.51	ns
Year 2	35.6a	32.1a	31.2a	29.38a	0.77	0.000	0.303	*Ŷ* = 37.0778 − 0.02*x*	0.93
Tiller population density (*n*)									
Year 1	458a	502a	506a	520b	17.68	ns	ns	*Ŷ* = 496.95	ns
Year 2	276b	404b	512a	565a	21.73	0.000	0.139	*Ŷ* = 196.15 + 1.29*x*	0.97

*Note:* Means followed by the same lowercase letter in the column were not different according to Tukey's test at a 5% significance level (*p* < 0.05).

Abbreviations: ns, not significant; *p*lin, *p*‐value for the linear effect; Post‐CH, post‐grazing canopy height; *p*quad, *p*‐value for the quadratic effect; Pre‐CH, pre‐grazing canopy height (cm); *R* ^2^, coefficient of determination; SEM, standard error of the mean.

For TPD, there was no effect of N doses in Year 1, with an average of 496 tillers/m^2^. In Year 2, the data fit the positive linear regression equation model, with an increase of 1.29 tillers for each kg N ha^−1^ year^−1^ added (Table 2). When the effect of experimental year was evaluated within each N fertilization dose, TPD decreased by 40% and 19% in the N75 and N150 treatments, respectively. In contrast, no change was observed in N225, whereas TPD increased by 8.6% in N300.

### 3.2 Forage Production, Morphological Components, and Nutritional Value

There was no interaction between N doses and year for the variables DM (*p* = 1.000), FMpre (*p* = 0.241), FMpost (*p* = 0.8437), FA (*p* = 0.0750) and FAR (*p* = 0.0801), but there was an isolated effect of N dose and year (*p* < 0.05) for all variables (Table 3), except for FMpost (*p* > 0.05; 3594 kg DM ha^−1^).

**TABLE 3:** Percentage of dry matter, pre‐ and post‐grazing forage mass, forage accumulation, and forage accumulation rates of Quênia guineagrass pastures in response to increasing doses of nitrogen fertilization.

Variable	Nitrogen doses (kg ha^−1^ year^−1^ of N)				SEM	*p*lin	*p*quad	Equation	*R* ^2^
	75	150	225	300					
DM (%)	28.6	25.9	25.5	25.4	0.7	0.004	0.075	*Ŷ* = 28.79 − 0.01*x*	0.71
FMpre (kg DM ha^−1^)	6202	6460	7316	7574	228.8	0.000	0.264	*Ŷ* = 5773.89 + 5.94*x*	0.75
FMpost (kg DM ha^−1^)	3860	3378	3577	3561	139.7	ns	ns	*Ŷ* = 3594.29	ns
FA (kg DM ha^−1^)	3143	3392	4179	4076	231.2	0.001	0.448	*Ŷ* = 2801.38 + 4.78*x*	0.82
FAR (kg DM ha^−1^ day^−1^)	71.2	96.8	159.3	168.8	13.0	0.000	0.537	*Ŷ* = 35.19 + 0.47*x*	0.93

Variable	Year		
	Year 1	Year 2	SEM
DM (%)	25.2	26.9	0.46
FMpre (kg DM ha^−1^)	6348	7671	148.53
FA (kg DM ha^−1^)	3407	4210	151.78
FAR (kg DM ha^−1^ day^−1^)	128	168	9.10

*Note:* Means followed by the same lowercase letter in the column were not significantly different according to Tukey's test at 5% significance (*p* < 0.05).

Abbreviations: DM, percentage of dry matter (%); FA, forage accumulation (kg DM ha^−1^); FAR, forage accumulation rate (kg DM ha^−1^ day^−1^); FMpost, post‐grazing forage mass (kg DM ha^−1^); FMpre, pre‐grazing forage mass (kg DM ha^−1^); ns, not significant; *p*lin, *p*‐value for the linear effect; *p*quad, *p*‐value for the quadratic effect; *R* ^2^, coefficient of determination; SEM, standard error of the mean.

The DM adjusted to the decreasing linear regression equation, with a decrease of 0.012% for each kg N ha^−1^ year^−1^ added (Table 3). The FMpre, FA, and FAR increased linearly with N doses, with increments of 5.94, 4.48, and 0.47 kg for each kg N ha^−1^ year^−1^ added.

There was an interaction between N dose and year for FP (*p* < 0.0001) and AEI (*p* < 0.0001). FP adjusted to the increasing linear regression equation model, with increments of 36.1 and 80.4 kg DM ha^−1^ year^−1^ in Year 1 and Year 2, respectively. When evaluating the effect of the year within each dose, a reduction in FP of 48.1% was observed from Year 1 to Year 2 for dose N75, and increases of 8.6%, 23.2%, and 48.5% for doses N150, N225, and N300 (Table 4). The AEI showed increases of 0.53% and 2.18% for each kg N ha^−1^ year^−1^ added in Year 1 and Year 2, respectively.

**TABLE 4:** Forage production and agronomic efficiency index of Quênia guineagrass pastures managed under increasing doses of nitrogen.

Year	Nitrogen doses (kg N ha^−1^ year^−1^)				SEM	*p*lin	*p*quad	Equation	*R* ^2^
	75	150	225	300					
FP (kg DM ha^−1^ year^−1^)									
Year 1	6960a	8622a	14,020b	14,181b	365.2	0.001	0.053	*Y* = 4180 + 36.17*x*	0.89
Year 2	3591b	9361a	17,267a	21,060a	365.2	< 0.001	0.54	*Y* = −2258.4 + 80.45*x*	0.99
AEI (%)									
Year 1	—	23.9b	101.5b	103.9b	10.5	< 0.001	—	*Y* = −43.3 + 0.53*x*	0.77
Year 2	—	160.7a	380.8a	486.5a	10.5	< 0.001	—	*Y* = −146.3 + 2.18*x*	0.96

*Note:* Means followed by the same lowercase letter in the column were not significantly different according to Tukey's test at 5% significance (*p* < 0.05).

Abbreviations: AE, agronomic efficiency index; FP, forage production; ns, not significant; *p*lin, *p*‐value for the linear effect; *p*quad, *p*‐value for the quadratic effect; *R* ^2^, coefficient of determination; SEM, standard error of the mean.

The AEI calculated using the FP of the N150 dose as a comparator parameter was 62.6 and 84.5 for N225, and AEI was 64.5 and 125.0 for N300, in Year 1 and Year 2, respectively. When the comparator parameter was the FP of the N225 dose, the AEI was 1.2% in Year 1 and 22% in Year 2 for the N300 dose.

### 3.3 Morphological Composition and Canopy Structure

There was no interaction between N doses and year for the morphological and structural composition of the Quênia guineagrass (*p* > 0.05); however, there was an isolated effect of N doses and/or year on all variables (Table 5). Regarding the effect of N doses, in the pre‐grazing condition, a linear increase was observed in the LP (*p* < 0.0001), LSR (*p* = 0.0470), and GDR (*p* < 0.0001) with estimated increments of 0.036%, 0.0001, and 0.0009 for each kg N ha^−1^ year^−1^ added, and a decrease of 0.038% in the percentage of DMPpre (*p* < 0.0001). In the post‐grazing, the data behavior was similar, with increases of 0.027%, 0.0002%, and 0.0021%, and a decrease of 0.0034% for the variables LPpost (*p* < 0.0001), LSRpost (0.0247%), GDRpost (0.0125), and DMPpost, respectively (Table 5).

**TABLE 5:** Leaf percentage, stem and dead material, leaf:stem ratio and green:dead ratio in pre‐ and post‐grazing of Quênia guineagrass as a function of increasing N doses, effect of evaluative year evaluated separately over 2 years.

Variable	Nitrogen doses (kg N ha^−1^ year^−1^)				SEM	*p*lin	*p*quad	Equation	*R* ^2^
	75	150	225	300					
LP_pre_	40.59	46.41	47.67	49.05	1.010	0.000	0.021	*Ŷ* = 39.26 + 0.03*x*	0.85
SP_pre_	34.01	32.76	33.86	34.28	0.884	ns	ns	*Ŷ* = 33.72	ns
DMP_pre_	25.40	20.83	18.46	16.67	0.849	0.000	0.071	*Ŷ* = 27.46 − 0.03*x*	0.94
LSR_pre_	1.22	1.44	1.44	1.46	0.052	0.011	0.041	*Ŷ* = 1.22 + 0.001*x*	0.72
GDR_pre_	3.09	3.91	4.71	5.50	0.243	0.00	0.494	*Ŷ* = 2.45 + 0.009*x*	0.91
LP_post_	13.54	17.56	18.85	19.99	0.82	0.000	0.084	*Ŷ* = 12.32 + 0.02*x*	0.89
SP_post_	41.52	42.08	42.63	42.90	1.46	ns	ns	*Ŷ* = 42.47	ns
DMP_post_	44.93	40.54	38.18	37.09	1.39	0.000	0.240	*Ŷ* = 46.65 − 0.03*x*	0.92
LSR_post_	0.34	0.45	0.47	0.49	0.031	0.002	0.191	*Ŷ* = 0.32 + 0.0002*x*	0.82
GDR_post_	1.29	1.49	1.66	1.78	0.103	0.001	0.670	*Ŷ* = 1.14 + 0.0021*x*	0.98

Variable	Year		
	Year 1	Year 2	SEM
LP_pre_	49.16	45.29	0.671
SP_pre_	31.72	35.37	0.586
LSR_pre_	1.56a	1.30b	0.039
LP_post_	21.50	15.65	0.541
SP_post_	37.04	47.01	0.967
DMP_post_	41.37	37.26	0.915
LSR_post_	0.59a	0.34b	0.022
GDR_post_	1.46b	1.75a	0.073

Abbreviations: DMPpost, percentage of dead material in post‐grazing (%); DMPpost, percentage of dead material in post‐grazing; GDRpost, green‐to‐dead ratio in post‐grazing; GDRpre, green‐to‐dead ratio in pre‐grazing; LPpost, percentage of leaves in pre‐grazing; LPpre, percentage of leaves in pre‐grazing (%); LSRpre, leaf‐to‐stem ratio in pre‐grazing; ns, not significant; *p*lin, *p*‐value for the linear effect; *p*quad, *p*‐value for the quadratic effect; *R* ^2^, coefficient of determination; SEM, standard error of the mean; SPpost, percentage of stem in post‐grazing; SPpre, percentage of stem in pre‐grazing.

When analyzing the year effect, a decrease was observed in LP (*p* = 0.0001) and LSRpre (*p* < 0.0001) in Year 2 and an increase for LPpre (*p* < 0.0001). In the post‐grazing condition, the behavior was similar, with the addition of the DMpost variable (*p* = 0.0020), which was higher in Year 1 (Table 5).

There was an interaction between N doses and year for crude protein (CP; *p* = 0.0044), neutral detergent fiber (NDF; *p* = 0.0004), and acid detergent fiber (ADF; *p* = 0.0029), and an isolated dose effect for organic matter digestibility (DIGOM; *p* = 0.0062) and lignin (LIG; *p* = 0.0045). CP increased linearly with N doses in both years, with a greater magnitude in Year 2. The year effect within each N dose showed a 24% decrease in CP in Year 2 for the N75 treatment, with no difference for the other treatments (Table 6).

**TABLE 6:** Nutritional value of the leaves of Quênia guineagrass pastures subjected to nitrogen fertilization and evaluated over 2 years.

Year	Nitrogen doses (kg N ha^−1^ year^−1^)				SEM	*p*lin	*p*quad	Equation	*R* ^2^
	75	150	225	300					
Crude protein (%)									
Year 1	12.22a	12.76a	13.11a	14.36a	0.457	0.001	0.653	*Ŷ* = 11.41 + 0.009*x*	0.92
Year 2	9.27b	11.90a	13.26a	14.68a	0.465	< 0.001	0.194	*Ŷ* = 7.88 + 0.002*x*	0.97
Neutral detergent fiber (%)									
Year 1	73.74b	72.41b	73.23	72.07	0.63	ns	ns	*Ŷ* = 72.86	ns
Year 2	78.89a	74.34a	72.86	72.72	0.643	< 0.001	0.036	*Ŷ* = 79.70 − 0.02*x*	0.79
Acid detergent fiber (%)									
Year 1	36.48b	35.98b	35.08b	33.99b	0.552	0.001	0.593	*Ŷ* = 37.47 − 0.01*x*	0.97
Year 2	40.75a	38.46a	37.16a	36.21a	0.561	< 0.001	0.239	*Ŷ* = 41.88 − 0.01*x*	0.95

Variable	Nitrogen doses (kg N ha^−1^ year^−1^)				SEM	*p*lin	*p*quad	Equation	*R* ^2^
	75	150	225	300					
DIGOM	53.36	56.26	58.22	59.86	1.305	< 0.001	0.630	*Ŷ* = 51.57 + 0.02*x*	0.98
LIG	3.32	3.78	3.69	3.51	0.001	0.797	0.0808	*Ŷ* = 4.05 − 0.001*x*	0.98

*Note:* Means followed by the same lowercase letter in the column were not significantly different according to Tukey's test at 5% significance (*p* < 0.05).

Abbreviations: DIGOM, Digestibility of organic matter; LIG, Lignin; ns, not significant; *p*lin, *p*‐value for the linear effect; *p*quad, *p*‐value for the quadratic effect; *R* ^2^, coefficient of determination; SEM, standard error of the mean.

The NDF and ADF decreased with increasing N doses (Table 6). In Year 1, there was no difference between the N doses for NDF (72.86%), and for ADF there was a decrease of 0.011% with the increase in N. In Year 2, the reduction was 0.026% for NDF and 0.019% for ADF for each kg N ha^−1^ year^−1^ added.

The DIGOM increased linearly with increasing N doses, with an increase of 0.028% for each kg N ha^−1^ year^−1^ added. LIG, on the other hand, showed a reduction of 0.0017% with increasing N doses (Table 6).

## 4 Discussion

The use of the 95% LI criterion as a management strategy allowed for standardizing the timing of animal entry and expressing the effects of N on growth dynamics and canopy structure, a condition associated with maximum net forage accumulation and the onset of losses due to internal shading (Da Silva et al. 2015).

In this context, the linear reduction in the grazing interval and consequently the increase in the number of GC with increasing N doses can be interpreted as an intensification strategy capable of increasing pasture use efficiency in the face of scenarios with greater climate variability (Ghahramani et al. 2019). Changes in rainfall patterns and the shortening of forage growth periods tend to accentuate the seasonality of production (Cedrez et al. 2023), especially in tropical regions, compromising forage availability throughout the year.

The greater number of GC in pastures located in tropical regions anticipates the entry of animals due to the effect of N on the acceleration of tissue flow in the plant (Braz et al. 2011), which alters the recovery rate of the photosynthetic apparatus (Cunha et al. 2022), decreasing the time required for the management goal to be achieved (95% LI) (Freitas et al. 2012). Thus, the increase in N doses allowed for greater use of pastures throughout the year. Pastures fertilized with N300 were used for 72 days longer than N75, 27 days longer than N150, and 16 days longer than N225.

Canopy height has been correlated with LI in several studies (Carnevalli et al. 2006; Barbosa et al. 2007; Alvarenga et al. 2020; Macedo et al. 2021). In this study, the pastures were managed equally (95% LI and a residue of 50% of the entry height); however, we found that as the N dose increased, the Quênia guineagrass reached the target of 95% IL at different heights (Table 2), and this change may be associated with changes caused by N in the morphological composition of the canopy (Table 5).

In this study, it was observed that, based on the average recommendation for nitrogen fertilization for the species *Panicum maximum* in production systems with a medium technological level (N150) (Gomide et al. 2016), the average pre‐grazing height corresponding to the 95% LI level was 65 cm (Table 3), a value close to that found by Jank et al. (2017) (height of 70 cm at 95% LI). The results indicate a possible flexibility in the pre‐grazing height for Quênia guineagrass in order to maximize the productive capacity of the forage, according to the nitrogen fertilization dose.

The linear increase in pre‐grazing FM, FA, and FP promoted by the intensification of N use confirms that N is the main limiting factor for productivity in intensively managed tropical pastures (Pereira Neto et al. 2024). However, the interpretation of these productive gains must be carried out in conjunction with the AEI, since increases in production do not necessarily imply greater nutrient use efficiency.

In the present study, although nitrogen fertilization increased FP in both years, marked increases in the AEI were observed only in the second year, particularly at the highest nitrogen rates. This response may reflect the cumulative effects of nitrogen fertilization on canopy structural adjustment and nutrient cycling over time (Zhang et al. 2024).

The canopy structure played a central role in mediating these effects. The increase in TDP at the highest N doses in the second year, associated with the reduction in canopy height, characterizes a size/density compensation process, in which smaller and more numerous tillers increase the efficiency of LI and the use of available resources (Sbrissia et al. 2020). Conversely, the significant reduction in TDP in pastures with lower fertilization (N75 and N150) shows a loss of structural persistence, indicating that N limitation compromises tiller renewal and the productive stability of the pasture over time (Carvalho et al. 2021).

Considering the high nutritional requirements of *Panicum maximum* cultivars, reduced levels of nitrogen fertilization, such as the dose of 75 kg N ha^−1^ year^−1^, were not sufficient to sustain tiller renewal (Table 3) and canopy persistence, favoring degradation processes over time (Euclides et al. 2014).

This scenario suggests that pastures managed under lower levels of nitrogen fertilization become more susceptible to environmental stresses, especially under conditions of climate variability (Figure 2), in which resource limitation can be intensified. In these situations, the reduced capacity for tiller renewal and canopy regeneration can compromise the system's resilience, reducing its ability to maintain production and persistence throughout the production cycles (Table 4). Thus, adequate nitrogen availability not only sustains productivity but also plays a fundamental role in maintaining the stability and sustainability of pastures under climate change scenarios (Escarcha et al. 2018; Henry et al. 2018).

The positive effect of N on changes in the morphological composition of the canopy can be observed with the increase in LSR in the pre‐grazing, which showed as its main factor the decrease in DMP as the dose of N increased (Table 5), even though this is a nutrient identified as an accelerator of senescence processes in plant tissues (Costa et al. 2013). The higher grazing frequency may have influenced the population of young tillers in the canopy, which, when managed correctly (95% LI), could be grazed before reaching high senescence rates.

The lower LP in the post‐grazing period of pastures fertilized with the N75 dose is related to a smaller remaining leaf area. Even using the same grazing intensity at all N doses, the greater canopy height observed for this dose (Table 2) may have altered the proportion of leaf area removal during grazing (Gastal and Lemaire 2015). This smaller leaf area requires the plant to mobilize more carbon and nitrogen reserves to quickly replenish the lost leaf area; however, the low level of light uptake by the remaining leaves can hinder this carbon mobilization and may affect the plant's ability to absorb N (Lemaire 2001). Thus, the lower light uptake associated with the low availability of N (N75) may have altered the rate of leaf area replenishment, resulting in higher GI of pastures fertilized with the N75 dose (Table 3).

This structural pattern was reinforced by changes in morphological composition, with an increase in the percentage of leaves, the leaf:stem ratio, and the green:dead ratio with increasing N doses, reflecting greater production of young tissues and a relative reduction in senescence. This effect is widely recognized as one of the main mechanisms by which N improves forage harvesting efficiency and animal performance (Waris et al. 2026). On the other hand, the reduction of these ratios in the second year, especially in the less fertilized treatments, indicates structural deterioration of the canopy, with a greater participation of stems and tissues of lower nutritional value.

The improvement in the nutritional value of the forage with the increase in N doses, evidenced by the increase in CP and DIGOM and the reduction in NDF and ADF levels, reinforces the role of N in the quality of the diet available to the animals. These results are in line with recent evidence demonstrating that nitrogen fertilization increases the concentration of soluble nitrogen compounds and reduces the structural fibrous fraction, favoring consumption and digestibility (Kamran et al. 2022; Lima et al. 2023). However, the sharp drop in CP in the second year for the N75 treatment and higher NDF and ADF contents may reflect the low availability of N, which led to the allocation of carbon to secondary cell wall components, such as cellulose and lignin (Waris et al. 2026) and shows that systems with low N availability can evolve into conditions of nutritional limitation, compromising animal performance and the efficiency of the production system (Delevatti et al. 2019). This shows that nitrogen fertilization also plays an important role in mitigating the negative effects of climatically unfavorable years on forage quality and system performance.

The integrated analysis between productivity and efficiency highlights a critical point for decision‐making: although the highest doses of N (N300) provided the highest values of FP and AEI, the marginal gain in relation to the intermediate dose (N225) was relatively small, especially in the second year. This behavior characterizes the occurrence of diminishing returns to nitrogen fertilization (Mitscherlich 1912), indicating that part of the additional N applied is not efficiently converted into forage (Martuscello et al. 2018).

Although greenhouse gas emissions were not measured in the present study, our findings may have important environmental implications when interpreted in light of previous research. Increased nitrogen fertilization has been consistently associated with higher emissions of nitrous oxide (N_2_O), one of the main greenhouse gases from agriculture, as well as greater nitrogen losses through volatilization and leaching (Manco et al. 2024; Tian et al. 2020). Consequently, highly fertilized systems are generally expected to have a greater environmental impact per unit area (Penuelas et al. 2023). However, previous studies have also shown that, when evaluated in terms of emission intensity (emissions per unit of product), more productive systems may exhibit improved environmental performance because emissions are distributed over a greater quantity of forage produced (Beauchemin et al. 2020; Waris et al. 2026).

Likewise, based on evidence from the literature, systems receiving lower nitrogen inputs, such as N75 in the present study, may generate lower absolute emissions but also lower productivity and resource‐use efficiency. This combination could result in higher emission intensity per unit of forage produced and may contribute to pasture degradation over time (Cardoso et al. 2020). Therefore, while these environmental implications were not directly assessed in this study, they suggest that optimizing nitrogen fertilization may improve the balance between forage productivity and environmental performance.

Strategies that seek to optimize the use of N, increasing its efficiency and reducing losses, are fundamental to making production systems more resilient and sustainable (Ludemann et al. 2024). Although higher doses of fertilizers can increase productivity and forage quality, calibration must consider financial and environmental objectives. In this context, the results obtained indicate that the adoption of intermediate doses of N may represent the most efficient strategy, balancing productivity, agronomic efficiency, adaptation to climate change, and environmental impact.

## 5 Conclusion

The reduced N dose (75 kg N ha^−1^ year^−1^) compromised forage production in Year 2 and increased the vulnerability of the system, indicating that 150 kg N ha^−1^ year^−1^ represents the minimum level to sustain the productivity and stability of Quênia guineagrass.

On the other hand, although high doses increase production, the gains in agronomic efficiency become marginal above 225 kg N ha^−1^ year^−1^, not justifying the higher economic costs and possible environmental impacts associated with higher doses. Thus, the range between 150 and 225 kg N ha^−1^ year^−1^ is the most suitable to reconcile productive intensification, economic viability and environmental sustainability, especially in scenarios of greater climatic variability.

## Funding

The authors have nothing to report.

## Conflicts of Interest

The authors declare no conflicts of interest.

## Supporting information


**Data S1:** Complete dataset used for the statistical analyses performed in this study.

## Data Availability

The data that supports the findings of this study are available in the Data S1.
