# Supplementary material for: Nitrogen‐Driven Changes in Canopy Structure Enhance Productivity and Efficiency of Guineagrass ( Megathyrsus maximus ) cv. BRS Quênia Across Contrasting Growing Seasons
Source: Plant Environ Interact. 2026 Oct 23;7(5):e70212. doi: 10.1002/pei3.70212 (PMC13691534; doi:10.1002/pei3.70212)
Supplement: Supplementary file 1 [file PEI3-7-e70212-s001.pdf]

| TRA | BL | AN | IL,N,4 | IEP | ACB,N,( | TAB,N | ACUMU | TAF,N, | DPP,N | ALTP | MS,N | MF,N,6, | FPRE | CPRE | MMPI | RFCF |
|---|---|---|---|---|---|---|---|---|---|---|---|---|---|---|---|---|
| 75 | 1 | 1 | 96,5 | 29 | 2281,1 | 78,7 | 1929,8 | 66,5 | 499,0 | 65,0 | 24,8 | 5119,8 | 37,0 | 28,8 | 34,3 | 1,28 |
| 75 | 2 | 1 | 94,6 | 22 | 866,3 | 39,4 | 2320,2 | 105,5 | 567,0 | 54,8 | 27,2 | 5543,3 | 47,9 | 28,9 | 23,1 | 1,66 |
| 75 | 4 | 1 | 94,9 | 29 | 1284,7 | 44,3 | 2131,0 | 73,5 | 514,0 | 57,9 | 28,9 | 5486,7 | 44,1 | 29,3 | 26,6 | 1,50 |
| 75 | 3 | 1 | 92,6 | 45 | 2307,0 | 61,0 | 2733,0 | 85,0 | 418,0 | 60,5 | 26,4 | 6316,0 | 46,1 | 35,7 | 18,2 | 1,29 |
| 75 | 1 | 1 | 94,9 | 34 | 2690,0 | 79,1 | 3198,7 | 94,1 | 430,0 | 58,2 | 23,7 | 4329,3 | 40,4 | 24,6 | 35,1 | 1,64 |
| 75 | 2 | 1 | 95,3 | 47 | 3970,7 | 84,5 | 4084,5 | 86,9 | 567,0 | 61,5 | 27,7 | 5881,0 | 42,1 | 38,7 | 19,3 | 1,09 |
| 150 | 2 | 1 | 95,5 | 29 | 2511,7 | 86,6 | 3258,1 | 112,3 | 573,0 | 65,3 | 23,4 | 5798,7 | 51,7 | 26,1 | 22,2 | 1,98 |
| 150 | 1 | 1 | 94,9 | 26 | 2083,0 | 80,1 | 2995,6 | 115,2 | 551,0 | 54,6 | 23,0 | 6251,0 | 43,2 | 26,6 | 30,2 | 1,62 |
| 150 | 3 | 1 | 94,2 | 20 | 1465,7 | 73,3 | 2572,9 | 128,6 | 352,0 | 56,8 | 24,2 | 5542,7 | 41,9 | 31,2 | 26,9 | 1,34 |
| 150 | 4 | 1 | 94,8 | 25 | 2431,0 | 103,0 | 3046,0 | 131,0 | 598,0 | 58,8 | 31,5 | 4530,7 | 43,9 | 32,4 | 23,7 | 1,36 |
| 150 | 1 | 1 | 94,5 | 20 | 2254,0 | 112,7 | 3195,6 | 159,8 | 490,0 | 54,6 | 28,1 | 6190,7 | 51,3 | 29,7 | 18,9 | 1,73 |
| 150 | 2 | 1 | 94,4 | 24 | 3118,0 | 129,9 | 2743,6 | 114,3 | 544,0 | 54,7 | 26,2 | 6710,0 | 50,3 | 30,3 | 19,4 | 1,70 |
| 150 | 3 | 1 | 94,8 | 21 | 3294,0 | 156,9 | 4457,2 | 212,2 | 427,0 | 56,7 | 27,1 | 6828,7 | 46,6 | 35,4 | 18,0 | 1,32 |
| 150 | 4 | 1 | 95,0 | 28 | 2289,7 | 81,8 | 3181,0 | 139,0 | 483,0 | 52,5 | 25,6 | 4933,3 | 52,8 | 25,7 | 21,4 | 2,05 |
| 225 | 2 | 1 | 94,5 | 34 | 3723,8 | 109,5 | 5240,3 | 154,1 | 489,0 | 57,4 | 26,7 | 5282,7 | 44,8 | 29,2 | 25,9 | 1,53 |
| 225 | 3 | 1 | 94,7 | 22 | 3939,8 | 179,1 | 4626,9 | 210,3 | 437,0 | 56,3 | 28,4 | 7796,0 | 54,5 | 32,5 | 13,0 | 1,67 |
| 225 | 1 | 1 | 95,0 | 23 | 2444,1 | 106,3 | 2109,7 | 91,7 | 439,0 | 69,8 | 28,0 | 8012,0 | 46,2 | 30,8 | 23,0 | 1,50 |
| 225 | 4 | 1 | 96,1 | 33 | 2444,1 | 74,1 | 2606,2 | 79,0 | 514,0 | 58,2 | 23,9 | 4974,7 | 58,6 | 24,8 | 16,6 | 2,36 |
| 225 | 2 | 1 | 96,2 | 23 | 3180,0 | 138,3 | 3160,4 | 137,4 | 441,0 | 54,1 | 24,3 | 5421,3 | 60,7 | 28,5 | 10,8 | 2,13 |
| 225 | 3 | 1 | 96,6 | 19 | 3238,0 | 170,4 | 3262,5 | 171,7 | 681,0 | 55,1 | 25,5 | 7436,0 | 36,1 | 34,2 | 29,7 | 1,05 |
| 225 | 1 | 1 | 94,5 | 33 | 2292,2 | 69,5 | 2648,5 | 80,3 | 478,0 | 58,2 | 29,2 | 7543,3 | 38,5 | 35,4 | 26,1 | 1,09 |
| 225 | 4 | 1 | 95,4 | 31 | 2292,2 | 73,9 | 2868,2 | 92,5 | 616,0 | 53,4 | 25,8 | 5890,7 | 50,9 | 34,9 | 14,2 | 1,46 |
| 225 | 2 | 1 | 95,2 | 24 | 3448,6 | 159,9 | 4430,1 | 184,6 | 483,0 | 49,7 | 27,8 | 7063,0 | 51,5 | 28,8 | 19,6 | 1,79 |
| 225 | 3 | 1 | 95,0 | 24 | 5146,0 | 214,4 | 4472,3 | 186,3 | 488,0 | 53,6 | 25,4 | 8953,3 | 40,0 | 30,8 | 29,3 | 1,30 |
| 300 | 1 | 1 | 95,5 | 29 | 2373,9 | 81,9 | 4722,0 | 162,8 | 538,0 | 66,8 | 29,9 | 7312,6 | 45,5 | 36,1 | 18,4 | 1,26 |
| 300 | 2 | 1 | 94,8 | 23 | 2179,0 | 94,7 | 2962,5 | 128,8 | 555,0 | 56,1 | 26,6 | 4962,0 | 54,5 | 33,6 | 11,9 | 1,62 |
| 300 | 3 | 1 | 94,6 | 30 | 2495,3 | 83,2 | 2896,6 | 96,6 | 548,0 | 56,8 | 26,9 | 5774,7 | 50,7 | 32,9 | 16,5 | 1,54 |
| 300 | 4 | 1 | 95,2 | 26 | 2705,3 | 104,1 | 3227,2 | 124,1 | 637,0 | 56,0 | 30,5 | 5949,3 | 45,2 | 41,1 | 13,7 | 1,10 |
| 300 | 1 | 1 | 94,9 | 17 | 4179,0 | 245,8 | 3314,2 | 195,0 | 525,0 | 59,4 | 24,7 | 7425,0 | 56,4 | 32,9 | 10,8 | 1,75 |
| 300 | 2 | 1 | 94,8 | 25 | 2264,7 | 90,6 | 3106,3 | 124,3 | 450,0 | 56,1 | 23,8 | 5296,7 | 57,6 | 33,4 | 9,0 | 1,72 |
| 300 | 3 | 1 | 95,8 | 28 | 2990,9 | 106,8 | 3346,0 | 119,5 | 488,0 | 55,7 | 27,4 | 5513,3 | 49,0 | 35,0 | 16,8 | 1,40 |
| 300 | 4 | 1 | 95,1 | 31 | 4382,7 | 141,4 | 4397,1 | 141,8 | 472,0 | 52,7 | 30,0 | 6922,7 | 49,8 | 36,6 | 13,5 | 1,36 |
| 300 | 1 | 1 | 95,0 | 24 | 3518,0 | 146,6 | 4123,6 | 171,8 | 513,0 | 55,7 | 24,2 | 7597,3 | 54,8 | 33,1 | 12,1 | 1,65 |
| 75 | 1 | 2 | 95,7 | 32 | 3678,0 | 114,9 | 3804,4 | 118,9 | 263,0 | 72,8 | 31,0 | 7182,0 | 46,6 | 23,8 | 29,6 | 1,95 |
| 75 | 4 | 2 | 95,2 | 35 | 3669,3 | 104,8 | 3840,5 | 109,7 | 313,0 | 71,8 | 30,7 | 7635,0 | 33,8 | 40,1 | 26,1 | 0,84 |
| 75 | 3 | 2 | 95,2 | 49 | 3598,0 | 73,4 | 3583,3 | 73,1 | 265,0 | 74,7 | 29,4 | 6752,3 | 41,1 | 32,2 | 26,8 | 1,28 |
| 75 | 2 | 2 | 94,7 | 47 | 4062,7 | 86,4 | 3692,3 | 78,6 | 265,0 | 71,6 | 35,9 | 7776,0 | 36,8 | 43,1 | 20,0 | 0,85 |
| 150 | 3 | 2 | 94,8 | 26 | 1171,0 | 45,0 | 2681,3 | 103,1 | 422,0 | 63,4 | 22,7 | 7020,0 | 45,9 | 34,8 | 19,2 | 1,32 |
| 150 | 4 | 2 | 94,9 | 31 | 3712,3 | 119,8 | 3977,7 | 128,3 | 400,0 | 63,1 | 22,2 | 7009,0 | 47,0 | 33,3 | 19,7 | 1,41 |
| 150 | 1 | 2 | 94,6 | 32 | 1994,7 | 62,3 | 2305,8 | 72,1 | 410,0 | 72,1 | 28,7 | 6934,0 | 47,7 | 29,7 | 22,6 | 1,61 |
| 150 | 2 | 2 | 95,0 | 32 | 1388,0 | 43,4 | 2044,9 | 63,9 | 313,0 | 70,1 | 26,1 | 6282,0 | 46,7 | 31,1 | 22,2 | 1,50 |
| 150 | 4 | 2 | 94,9 | 28 | 2374,2 | 84,8 | 5324,5 | 190,2 | 485,0 | 59,9 | 23,2 | 6478,7 | 39,1 | 43,1 | 17,8 | 0,91 |
| 150 | 1 | 2 | 94,9 | 32 | 5072,0 | 158,5 | 4849,2 | 151,5 | 421,0 | 65,4 | 27,7 | 8586,7 | 45,1 | 35,9 | 19,0 | 1,26 |
| 150 | 2 | 2 | 95,5 | 32 | 3666,0 | 114,6 | 4259,2 | 133,1 | 383,0 | 62,1 | 29,3 | 7807,0 | 37,8 | 47,4 | 14,8 | 0,80 |
| 225 | 2 | 2 | 94,7 | 18 | 3725,7 | 207,0 | 4672,3 | 259,6 | 519,0 | 66,8 | 25,9 | 7847,3 | 49,7 | 42,5 | 17,7 | 1,17 |
| 225 | 3 | 2 | 94,8 | 26 | 5038,1 | 193,8 | 5485,0 | 211,0 | 418,0 | 59,1 | 30,0 | 7446,7 | 50,1 | 37,4 | 12,4 | 1,34 |
| 225 | 4 | 2 | 95,4 | 27 | 4457,3 | 165,1 | 5454,8 | 202,0 | 490,0 | 68,3 | 24,0 | 8153,0 | 50,5 | 32,3 | 17,2 | 1,56 |
| 225 | 1 | 2 | 95,2 | 33 | 4169,3 | 126,3 | 5502,2 | 166,7 | 492,0 | 65,2 | 22,0 | 8202,7 | 50,1 | 37,4 | 12,4 | 1,34 |
| 225 | 2 | 2 | 96,8 | 29 | 3998,0 | 137,9 | 4223,3 | 145,6 | 427,0 | 60,0 | 22,7 | 8320,7 | 38,4 | 44,0 | 17,7 | 0,87 |
| 225 | 3 | 2 | 94,6 | 19 | 5417,3 | 285,1 | 5329,0 | 280,5 | 488,0 | 58,9 | 21,9 | 7901,3 | 54,2 | 28,7 | 17,0 | 1,89 |
| 225 | 4 | 2 | 95,0 | 23 | 5372,7 | 233,6 | 4673,3 | 203,2 | 532,0 | 64,0 | 22,0 | 8198,0 | 44,5 | 39,7 | 15,7 | 1,12 |
| 225 | 1 | 2 | 95,0 | 26 | 5294,0 | 203,6 | 4832,0 | 185,8 | 436,0 | 64,7 | 23,3 | 8018,7 | 39,0 | 38,0 | 23,0 | 1,03 |
| 225 | 2 | 2 | 94,8 | 26 | 5567,3 | 214,1 | 4832,4 | 185,9 | 495,0 | 57,1 | 32,0 | 8473,0 | 45,2 | 39,5 | 15,3 | 1,14 |
| 225 | 3 | 2 | 95,7 | 26 | 5134,1 | 197,5 | 4649,8 | 178,8 | 480,0 | 63,5 | 23,5 | 8076,0 | 40,4 | 37,6 | 12,0 | 1,08 |

| | | | | | | | | | | | | | | | | |
|---|---|---|---|---|---|---|---|---|---|---|---|---|---|---|---|---|
| 225 | 4 | 2 | 94,9 | 23 | 4746,7 | 206,4 | 4320,2 | 187,8 | 570,0 | 66,2 | 21,6 | 8702,0 | 38,8 | 40,4 | 20,8 | 0,96 |
| 225 | 1 | 2 | 95,0 | 37 | 5559,0 | 150,2 | 4686,3 | 126,7 | 549,0 | 58,2 | 22,0 | 7576,7 | 39,3 | 41,2 | 19,5 | 0,95 |
| 300 | 1 | 2 | 95,3 | 19 | 5980,0 | 314,7 | 6289,5 | 331,0 | 532,0 | 62,3 | 23,6 | 7831,1 | 45,8 | 35,7 | 18,5 | 1,32 |
| 300 | 2 | 2 | 95,5 | 17 | 5068,7 | 298,2 | 5576,1 | 328,0 | 547,0 | 64,1 | 25,9 | 8606,0 | 50,5 | 32,3 | 17,2 | 1,56 |
| 300 | 3 | 2 | 95,8 | 16 | 5312,7 | 265,6 | 5753,7 | 287,7 | 591,0 | 58,0 | 32,9 | 9451,3 | 46,1 | 35,5 | 18,4 | 1,32 |
| 300 | 4 | 2 | 95,2 | 20 | 5614,7 | 350,9 | 6045,9 | 377,9 | 655,0 | 58,1 | 23,6 | 7831,1 | 46,1 | 35,5 | 18,4 | 1,32 |
| 300 | 1 | 2 | 94,9 | 19 | 4366,7 | 229,8 | 3576,3 | 188,2 | 570,0 | 60,5 | 25,3 | 8062,7 | 48,9 | 29,6 | 21,5 | 1,65 |
| 300 | 2 | 2 | 96,3 | 24 | 4465,3 | 186,1 | 4382,9 | 182,6 | 527,0 | 58,7 | 22,1 | 8219,3 | 46,8 | 37,5 | 15,7 | 1,25 |
| 300 | 3 | 2 | 95,3 | 30 | 3104,0 | 103,5 | 2493,7 | 176,0 | 568,0 | 61,4 | 24,3 | 7169,3 | 38,6 | 32,7 | 28,6 | 1,18 |
| 300 | 4 | 2 | 95,3 | 22 | 3996,9 | 181,7 | 3452,4 | 156,9 | 569,0 | 60,0 | 26,6 | 7835,3 | 47,7 | 30,2 | 22,2 | 1,58 |
| 300 | 1 | 2 | 94,9 | 17 | 2814,0 | 165,5 | 4280,0 | 251,8 | 466,0 | 62,2 | 20,5 | 7165,3 | 47,4 | 41,0 | 11,6 | 1,16 |
| 300 | 2 | 2 | 96,5 | 18 | 2795,3 | 155,3 | 4195,4 | 233,1 | 583,0 | 57,5 | 22,7 | 6899,9 | 58,1 | 32,2 | 9,7 | 1,80 |
| 300 | 3 | 2 | 95,3 | 27 | 2896,0 | 107,3 | 3907,9 | 144,7 | 591,0 | 59,9 | 26,7 | 6899,9 | 37,7 | 45,9 | 16,4 | 0,82 |
| 300 | 4 | 2 | 95,3 | 18 | 3188,0 | 177,1 | 3681,5 | 204,5 | 534,0 | 59,9 | 22,4 | 7526,7 | 46,5 | 37,5 | 15,9 | 1,24 |
| 300 | 1 | 2 | 95,8 | 24 | 6368,7 | 265,4 | 5228,4 | 217,9 | 668,0 | 60,0 | 20,1 | 8796,0 | 43,6 | 33,9 | 22,4 | 1,29 |
| 300 | 2 | 2 | 95,0 | 23 | 4431,3 | 192,7 | 3751,3 | 163,1 | 502,0 | 55,7 | 18,6 | 7093,3 | 42,2 | 36,7 | 21,0 | 1,15 |
| 300 | 3 | 2 | 95,2 | 44 | 4752,8 | 108,0 | 4559,6 | 103,6 | 469,0 | 52,7 | 20,5 | 8103,8 | 43,8 | 36,1 | 20,1 | 1,21 |
| 300 | 4 | 2 | 96,0 | 24 | 4530,0 | 188,7 | 4780,7 | 199,2 | 480,0 | 59,7 | 22,9 | 8422,0 | 45,6 | 37,6 | 16,8 | 1,21 |
| 300 | 2 | 2 | 95,3 | 39 | 4341,3 | 111,3 | 3887,6 | 99,7 | 553,0 | 58,2 | 23,0 | 7216,0 | 43,6 | 36,9 | 19,5 | 1,18 |
| 300 | 2 | 1 | 95,5 | 26 | 4898,7 | 188,4 | 5834,3 | 224,4 | 478,0 | 53,0 | 30,3 | 8218,0 | 50,6 | 40,0 | 9,4 | 1,26 |
| 300 | 1 | 1 | 94,2 | 35 | 6073,3 | 173,5 | 4200,7 | 120,0 | 522,0 | 55,7 | 33,8 | 9401,3 | 27,4 | 39,7 | 32,9 | 0,69 |
| 225 | 3 | 2 | 93,7 | 39 | 5112,8 | 131,1 | 4041,1 | 103,6 | 641,0 | 61,0 | 28,3 | 8680,7 | 40,7 | 35,9 | 23,3 | 1,13 |
| 225 | 1 | 2 | 94,5 | 25 | 3596,7 | 143,9 | 2177,3 | 87,1 | 595,0 | 58,6 | 25,2 | 7384,7 | 32,6 | 34,6 | 32,8 | 0,94 |
| 225 | 4 | 2 | 95,7 | 53 | 4928,0 | 93,0 | 4188,2 | 79,0 | 548,0 | 60,0 | 28,2 | 8006,7 | 38,2 | 42,3 | 19,5 | 0,90 |
| 300 | 4 | 2 | 95,3 | 39 | 3198,0 | 82,0 | 3592,1 | 92,1 | 635,0 | 55,5 | 27,1 | 6778,7 | 46,5 | 41,8 | 11,7 | 1,11 |
| 300 | 1 | 2 | 94,5 | 23 | 4013,3 | 174,5 | 2273,2 | 98,8 | 642,0 | 56,1 | 25,9 | 7065,3 | 36,2 | 30,3 | 33,5 | 1,20 |
| 300 | 2 | 2 | 94,9 | 28 | 3316,4 | 118,4 | 2530,1 | 90,4 | 629,0 | 56,5 | 26,9 | 5472,0 | 40,3 | 33,3 | 26,4 | 1,21 |

| RVMPI | MASFO | COLMA | MSMMF | ALTPC | MSPO: | MFPOS,I | FOLPC | CPOS, | MMPO | RFCF | RVMI | MASFOL |
|---|---|---|---|---|---|---|---|---|---|---|---|---|
| 1,92 | 1892,5 | 1473,7 | 1753,5 | 32,00 | 23,99 | 3626,00 | 20,94 | 33,25 | 45,80 | 0,63 | 1,18 | 759,38 |
| 3,32 | 2656,5 | 1604,6 | 1282,3 | 26,40 | 28,80 | 3279,33 | 24,25 | 29,68 | 46,08 | 0,82 | 1,17 | 795,08 |
| 2,76 | 2418,8 | 1607,6 | 1460,2 | 29,50 | 27,62 | 3771,33 | 26,38 | 39,06 | 34,55 | 0,68 | 1,89 | 994,94 |
| 4,48 | 2909,7 | 2254,2 | 1152,1 | 28,80 | 33,44 | 4811,00 | 22,22 | 29,59 | 48,19 | 0,75 | 1,08 | 1068,97 |
| 1,85 | 1748,0 | 1063,8 | 1517,5 | 30,80 | 32,48 | 3529,33 | 23,63 | 42,68 | 33,69 | 0,55 | 1,97 | 833,94 |
| 4,19 | 2473,1 | 2274,5 | 1133,4 | 29,60 | 34,08 | 4464,67 | 16,60 | 35,65 | 47,75 | 0,47 | 1,09 | 741,10 |
| 3,50 | 2997,3 | 1513,6 | 1287,8 | 33,80 | 28,63 | 3592,00 | 21,21 | 36,94 | 41,85 | 0,57 | 1,39 | 761,70 |
| 2,32 | 2700,2 | 1665,8 | 1885,0 | 27,50 | 22,33 | 3936,67 | 20,48 | 25,86 | 53,66 | 0,79 | 0,86 | 806,39 |
| 2,71 | 2320,9 | 1728,4 | 1493,4 | 27,60 | 29,85 | 3534,67 | 13,60 | 18,75 | 67,65 | 0,73 | 0,48 | 480,67 |
| 3,23 | 1990,1 | 1468,4 | 1072,1 | 28,40 | 33,02 | 3876,00 | 13,14 | 43,23 | 43,63 | 0,30 | 1,29 | 509,28 |
| 4,29 | 3178,3 | 1841,5 | 1170,8 | 27,90 | 30,54 | 2970,00 | 30,34 | 46,29 | 23,37 | 0,66 | 3,28 | 901,08 |
| 4,17 | 2989,4 | 1842,7 | 1152,1 | 26,70 | 29,78 | 3027,33 | 31,77 | 34,20 | 34,03 | 0,93 | 1,94 | 961,73 |
| 4,56 | 3184,0 | 2416,7 | 1227,9 | 28,90 | 25,95 | 2934,00 | 26,64 | 38,08 | 35,28 | 0,70 | 1,83 | 781,64 |
| 3,67 | 2606,0 | 1269,9 | 1057,5 | 26,90 | 26,42 | 2636,00 | 33,15 | 40,28 | 26,58 | 0,82 | 2,76 | 873,79 |
| 2,86 | 2368,0 | 1544,6 | 1370,0 | 28,70 | 31,28 | 4256,00 | 17,45 | 31,09 | 51,46 | 0,56 | 0,94 | 742,81 |
| 6,69 | 4245,6 | 2536,5 | 1013,9 | 33,00 | 25,96 | 4305,33 | 13,31 | 40,38 | 46,31 | 0,33 | 1,16 | 573,09 |
| 3,35 | 3698,8 | 2469,9 | 1843,4 | 28,20 | 28,62 | 4280,67 | 15,38 | 35,74 | 48,88 | 0,45 | 1,05 | 658,46 |
| 5,02 | 2912,7 | 1235,2 | 826,8 | 26,30 | 28,62 | 4280,67 | 15,38 | 35,74 | 48,88 | 0,45 | 1,05 | 658,46 |
| 8,27 | 3290,8 | 1545,9 | 584,6 | 27,90 | 28,65 | 4761,33 | 20,91 | 31,22 | 47,87 | 0,67 | 1,09 | 995,80 |
| 2,37 | 2681,8 | 2544,6 | 2209,5 | 29,20 | 29,80 | 3807,33 | 19,80 | 29,07 | 51,13 | 0,68 | 0,96 | 753,98 |
| 2,83 | 2902,4 | 2671,8 | 1969,1 | 27,60 | 26,29 | 3082,00 | 24,50 | 48,10 | 27,41 | 0,51 | 2,65 | 754,94 |
| 6,06 | 2998,2 | 2058,3 | 834,3 | 24,70 | 32,90 | 3954,00 | 17,27 | 31,23 | 51,49 | 0,55 | 0,94 | 682,98 |
| 4,10 | 3639,3 | 2037,7 | 1386,0 | 25,30 | 29,82 | 2939,33 | 19,83 | 39,36 | 40,80 | 0,50 | 1,45 | 582,99 |
| 2,42 | 3578,4 | 2754,5 | 2620,5 | 26,40 | 30,16 | 3558,00 | 17,93 | 47,18 | 34,89 | 0,38 | 1,87 | 637,89 |
| 4,44 | 3325,7 | 2641,7 | 1345,1 | 34,00 | 29,64 | 3246,00 | 19,01 | 48,10 | 32,88 | 0,40 | 2,04 | 617,17 |
| 7,41 | 2706,0 | 1666,2 | 589,8 | 28,50 | 30,14 | 3032,00 | 13,78 | 42,75 | 43,47 | 0,32 | 1,30 | 417,76 |
| 5,08 | 2925,9 | 1898,6 | 950,2 | 28,10 | 27,79 | 2540,00 | 19,41 | 43,13 | 37,46 | 0,45 | 1,67 | 493,12 |
| 6,30 | 2686,7 | 2447,7 | 814,9 | 26,00 | 36,23 | 4376,00 | 19,41 | 43,13 | 37,46 | 0,45 | 1,67 | 849,56 |
| 8,57 | 3459,7 | 2033,1 | 651,3 | 29,60 | 22,50 | 4079,33 | 24,11 | 38,60 | 37,29 | 0,62 | 1,68 | 983,51 |
| 10,12 | 3050,0 | 1770,3 | 476,3 | 31,00 | 24,96 | 3319,33 | 24,82 | 23,81 | 51,37 | 1,04 | 0,95 | 823,71 |
| 5,00 | 3399,8 | 1572,9 | 540,7 | 26,30 | 29,58 | 3964,00 | 22,87 | 38,56 | 38,57 | 0,59 | 1,59 | 906,42 |
| 6,39 | 3450,8 | 2534,8 | 937,0 | 26,10 | 30,88 | 4196,00 | 22,87 | 38,56 | 38,57 | 0,59 | 1,59 | 959,47 |
| 7,30 | 4164,3 | 2517,5 | 915,5 | 29,80 | 23,31 | 3328,00 | 21,02 | 42,39 | 36,59 | 0,50 | 1,73 | 699,44 |
| 2,38 | 3565,9 | 1824,9 | 2267,3 | 35,50 | 29,42 | 4325,33 | 7,48 | 43,48 | 49,04 | 0,17 | 1,04 | 323,48 |
| 2,83 | 2669,8 | 3162,9 | 2061,3 | 34,80 | 24,40 | 3174,67 | 9,74 | 58,86 | 31,40 | 0,17 | 2,19 | 309,22 |
| 2,74 | 2959,2 | 2317,6 | 1928,6 | 36,20 | 30,10 | 3250,00 | 8,23 | 51,39 | 40,38 | 0,16 | 1,48 | 267,51 |
| 3,99 | 2862,9 | 3354,9 | 1558,1 | 36,20 | 38,89 | 4370,00 | 8,21 | 40,26 | 51,53 | 0,20 | 0,94 | 358,80 |
| 4,20 | 3051,4 | 2313,1 | 1276,5 | 31,15 | 31,43 | 4104,44 | 11,45 | 46,36 | 42,19 | 0,26 | 1,37 | 470,01 |
| 4,09 | 3430,7 | 2002,9 | 581,4 | 35,10 | 23,39 | 3328,00 | 7,01 | 51,81 | 41,18 | 0,14 | 1,43 | 233,13 |
| 3,42 | 3310,0 | 2056,2 | 1567,7 | 33,90 | 27,91 | 4141,33 | 10,96 | 46,83 | 42,20 | 0,23 | 1,37 | 454,01 |
| 3,50 | 2932,6 | 1954,0 | 1395,4 | 31,45 | 26,23 | 3148,89 | 11,44 | 50,67 | 37,89 | 0,23 | 1,64 | 360,32 |
| 4,61 | 2533,2 | 2791,4 | 1154,1 | 29,80 | 24,96 | 2411,33 | 11,76 | 49,06 | 39,18 | 0,24 | 1,55 | 283,54 |
| 4,27 | 3875,2 | 3082,6 | 1628,8 | 32,30 | 24,37 | 2978,00 | 11,16 | 49,99 | 38,85 | 0,22 | 1,57 | 332,34 |
| 5,76 | 2619,7 | 3286,9 | 1024,7 | 31,60 | 29,37 | 4057,33 | 11,41 | 52,95 | 35,64 | 0,22 | 1,81 | 462,93 |
| 5,20 | 3903,0 | 3337,5 | 606,9 | 28,75 | 29,68 | 3903,33 | 13,22 | 46,57 | 40,21 | 0,28 | 1,49 | 515,98 |
| 7,04 | 3733,0 | 2785,8 | 926,4 | 35,65 | 27,00 | 2780,67 | 14,68 | 42,10 | 43,22 | 0,35 | 1,31 | 408,22 |
| 4,83 | 4349,4 | 2779,6 | 1477,0 | 35,25 | 29,25 | 2646,00 | 14,68 | 42,10 | 43,22 | 0,35 | 1,31 | 388,45 |
| 7,04 | 4112,0 | 3068,6 | 1020,4 | 33,70 | 26,40 | 3026,67 | 17,76 | 48,88 | 33,36 | 0,36 | 2,00 | 537,64 |
| 4,65 | 3191,9 | 3657,2 | 1471,6 | 30,55 | 20,58 | 3432,00 | 19,65 | 44,11 | 36,23 | 0,45 | 1,76 | 674,48 |
| 4,88 | 4286,1 | 2271,2 | 1344,1 | 33,05 | 27,00 | 3567,90 | 26,01 | 36,81 | 37,18 | 0,71 | 1,69 | 928,08 |
| 5,35 | 3652,1 | 3255,8 | 1290,1 | 31,40 | 26,95 | 2830,00 | 26,01 | 36,81 | 37,18 | 0,71 | 1,69 | 736,14 |
| 3,35 | 3130,6 | 3045,2 | 1842,9 | 29,65 | 23,68 | 2914,00 | 12,43 | 57,49 | 30,08 | 0,22 | 2,32 | 362,16 |
| 5,54 | 3153,3 | 3570,5 | 1749,2 | 29,65 | 25,38 | 3478,19 | 20,76 | 52,56 | 26,68 | 0,41 | 2,75 | 721,90 |
| 6,51 | 3640,2 | 3380,7 | 1978,4 | 34,10 | 27,00 | 3567,90 | 25,05 | 48,21 | 26,74 | 0,52 | 2,74 | 893,64 |

| | | | | | | | | | | | | |
|---|---|---|---|---|---|---|---|---|---|---|---|---|
| 3,81 | 3377,9 | 3513,5 | 1810,7 | 29,20 | 23,62 | 3078,67 | 25,05 | 48,21 | 26,74 | 0,52 | 2,74 | 771,10 |
| 4,12 | 2976,9 | 3121,2 | 1478,6 | 27,45 | 25,51 | 3788,00 | 12,17 | 61,26 | 26,57 | 0,20 | 2,76 | 461,07 |
| 4,81 | 3548,9 | 2755,4 | 1434,6 | 34,05 | 22,56 | 3754,00 | 23,70 | 44,10 | 32,20 | 0,54 | 2,11 | 889,70 |
| 4,83 | 4349,4 | 2779,6 | 1477,0 | 27,65 | 31,15 | 3838,44 | 24,30 | 44,61 | 31,09 | 0,54 | 2,28 | 932,66 |
| 4,82 | 3625,4 | 2774,0 | 1446,3 | 28,00 | 33,44 | 4065,33 | 21,87 | 42,62 | 35,51 | 0,51 | 1,82 | 889,04 |
| 4,82 | 3625,4 | 2774,0 | 1446,3 | 30,80 | 37,44 | 3696,00 | 27,32 | 47,11 | 25,56 | 0,58 | 2,91 | 1009,92 |
| 3,65 | 3941,5 | 2386,0 | 1735,2 | 30,05 | 23,87 | 2722,00 | 17,14 | 57,56 | 25,30 | 0,30 | 2,95 | 466,57 |
| 5,37 | 3846,2 | 3081,9 | 1291,2 | 30,45 | 24,60 | 2898,00 | 16,38 | 54,54 | 29,07 | 0,30 | 2,44 | 474,83 |
| 2,49 | 2769,5 | 2345,8 | 2054,0 | 31,10 | 26,29 | 4338,67 | 18,49 | 42,47 | 39,04 | 0,44 | 1,56 | 802,37 |
| 3,51 | 3734,7 | 2362,7 | 1738,0 | 30,50 | 22,61 | 3356,00 | 18,49 | 42,47 | 39,04 | 0,44 | 1,56 | 620,64 |
| 7,63 | 3397,8 | 2937,6 | 829,9 | 28,90 | 18,29 | 2662,00 | 15,34 | 54,15 | 30,51 | 0,28 | 2,28 | 408,36 |
| 9,28 | 4005,7 | 2222,9 | 671,3 | 30,00 | 19,86 | 2427,33 | 15,20 | 50,56 | 34,24 | 0,30 | 1,92 | 368,96 |
| 5,09 | 2684,4 | 3269,4 | 1169,2 | 31,10 | 23,26 | 3892,00 | 10,72 | 46,54 | 42,74 | 0,23 | 1,34 | 417,21 |
| 5,27 | 3502,5 | 2823,9 | 1200,3 | 30,00 | 24,50 | 3351,00 | 10,72 | 46,54 | 42,74 | 0,23 | 1,34 | 359,21 |
| 3,46 | 3838,6 | 2986,2 | 1971,3 | 28,00 | 20,26 | 2874,67 | 13,82 | 53,09 | 33,08 | 0,26 | 2,02 | 397,41 |
| 3,75 | 2995,4 | 2605,6 | 1492,3 | 28,50 | 23,78 | 3668,00 | 13,82 | 53,09 | 33,08 | 0,26 | 2,02 | 507,08 |
| 3,98 | 3557,8 | 2920,5 | 1625,5 | 30,70 | 21,52 | 3580,67 | 13,82 | 53,09 | 33,08 | 0,26 | 2,02 | 495,01 |
| 4,96 | 3839,3 | 3169,8 | 1412,8 | 27,00 | 29,55 | 4548,67 | 13,82 | 53,09 | 33,08 | 0,26 | 2,02 | 628,83 |
| 4,14 | 3145,8 | 2665,5 | 1404,7 | 28,40 | 28,84 | 4356,67 | 15,16 | 47,53 | 37,30 | 0,32 | 1,68 | 660,68 |
| 9,68 | 4158,0 | 3290,4 | 769,7 | 25,50 | 31,00 | 3591,33 | 16,00 | 55,38 | 28,62 | 0,29 | 2,49 | 574,61 |
| 2,04 | 2580,3 | 3730,6 | 3090,4 | 26,60 | 35,91 | 4581,00 | 15,24 | 44,55 | 40,20 | 0,34 | 1,49 | 698,37 |
| 3,29 | 3536,0 | 3119,0 | 2025,7 | 31,40 | 27,00 | 3567,90 | 7,86 | 42,59 | 49,54 | 0,18 | 1,02 | 280,60 |
| 2,04 | 2406,7 | 2552,4 | 2425,6 | 29,35 | 29,68 | 3669,33 | 15,27 | 45,28 | 39,45 | 0,34 | 1,53 | 560,31 |
| 4,12 | 3055,7 | 3387,9 | 1563,0 | 29,80 | 26,97 | 3966,67 | 7,86 | 42,59 | 49,54 | 0,18 | 1,02 | 311,96 |
| 7,58 | 3153,0 | 2835,3 | 790,4 | 27,70 | 25,77 | 2906,00 | 18,10 | 52,90 | 28,90 | 0,34 | 2,46 | 525,99 |
| 1,98 | 2559,7 | 2138,3 | 2367,4 | 27,05 | 32,22 | 3558,67 | 14,20 | 40,36 | 45,44 | 0,35 | 1,20 | 505,31 |
| 2,79 | 2205,9 | 1822,7 | 1443,4 | 27,65 | 31,31 | 3648,00 | 14,20 | 40,36 | 45,44 | 0,35 | 1,20 | 518,00 |

| MSACOL | MSSMMF | MSFOI | CINZA | PBFOI | FDNF( | FDAF( | DIGM( | LIGSI | CELF( | SILFOLHA,N,4,2 |
|---|---|---|---|---|---|---|---|---|---|---|
| 1205,82 | 1660,80 | 90,98 | 12,23 | 11,99 | 74,24 | 37,89 | 60,16 | 3,68 | 32,69 | 3,11 |
| 973,16 | 1511,09 | 90,59 | 13,32 | 13,75 | 72,89 | 36,63 | 56,94 | 3,94 | 31,92 | 3,85 |
| 1473,21 | 1303,18 | 90,93 | 11,35 | 11,69 | 73,03 | 36,40 | 57,47 | 3,67 | 31,56 | 2,69 |
| 1423,56 | 2318,47 | 90,86 | 11,87 | 13,30 | 72,76 | 35,65 | 59,75 | 3,71 | 31,55 | 2,85 |
| 1506,30 | 1189,10 | 89,23 | 12,39 | 12,20 | 75,83 | 36,50 | 57,70 | 4,71 | 34,90 | 3,00 |
| 1591,76 | 2131,80 | 91,47 | 10,58 | 10,43 | 73,72 | 35,82 | 54,38 | 3,67 | 30,88 | 2,63 |
| 1326,87 | 1503,42 | 90,47 | 11,41 | 13,36 | 73,46 | 35,48 | 59,39 | 3,64 | 31,51 | 2,62 |
| 1017,90 | 2112,38 | 88,28 | 13,52 | 11,34 | 71,05 | 36,00 | 57,03 | 4,31 | 33,03 | 2,80 |
| 662,85 | 2391,15 | 89,55 | 12,32 | 12,56 | 74,91 | 37,43 | 56,42 | 4,30 | 32,72 | 3,29 |
| 1675,57 | 1691,15 | 90,22 | 12,14 | 12,98 | 71,34 | 37,39 | 59,57 | 3,89 | 32,01 | 3,20 |
| 1374,79 | 694,13 | 90,39 | 11,75 | 12,93 | 72,40 | 36,82 | 58,65 | 3,82 | 31,32 | 2,74 |
| 1035,41 | 1030,19 | 90,47 | 11,41 | 13,36 | 73,46 | 35,48 | 59,39 | 3,64 | 31,51 | 2,62 |
| 1117,13 | 1035,22 | 91,47 | 12,05 | 12,80 | 71,62 | 33,31 | 63,23 | 3,36 | 30,22 | 2,35 |
| 1061,65 | 700,56 | 91,55 | 11,10 | 12,80 | 71,10 | 36,00 | 59,10 | 2,93 | 31,80 | 2,80 |
| 1323,23 | 2189,97 | 92,49 | 10,44 | 11,01 | 73,20 | 36,83 | 57,00 | 3,59 | 31,50 | 1,95 |
| 1738,63 | 1993,61 | 89,96 | 12,43 | 10,90 | 73,01 | 35,10 | 56,75 | 4,16 | 32,73 | 3,44 |
| 1529,78 | 2092,43 | 90,15 | 12,07 | 15,27 | 69,99 | 33,17 | 64,84 | 3,53 | 29,56 | 2,65 |
| 1529,78 | 2092,43 | 89,79 | 12,10 | 13,19 | 74,22 | 35,10 | 57,60 | 4,30 | 33,00 | 3,53 |
| 1486,42 | 2279,12 | 90,19 | 12,21 | 13,77 | 71,97 | 36,37 | 62,87 | 3,83 | 32,35 | 2,96 |
| 1106,63 | 1946,72 | 89,56 | 11,62 | 10,62 | 75,06 | 37,38 | 55,44 | 3,78 | 32,19 | 2,97 |
| 1482,31 | 844,75 | 91,89 | 10,79 | 12,55 | 73,20 | 36,46 | 57,93 | 4,06 | 31,50 | 1,87 |
| 1235,00 | 2036,02 | 92,43 | 11,40 | 15,91 | 73,17 | 31,76 | 66,69 | 3,13 | 30,60 | 1,62 |
| 1156,99 | 1199,35 | 92,72 | 12,30 | 14,20 | 74,19 | 33,97 | 60,80 | 3,52 | 31,51 | 2,57 |
| 1678,72 | 1241,39 | 91,41 | 10,62 | 13,73 | 74,30 | 34,68 | 57,83 | 3,79 | 31,47 | 2,22 |
| 1561,45 | 1067,37 | 90,92 | 11,37 | 12,23 | 71,35 | 36,25 | 61,49 | 3,40 | 31,38 | 2,59 |
| 1296,25 | 1317,98 | 90,09 | 13,09 | 15,62 | 72,13 | 34,53 | 59,89 | 3,82 | 31,36 | 2,50 |
| 1095,43 | 951,45 | 91,20 | 12,37 | 12,72 | 71,01 | 36,32 | 61,80 | 3,50 | 31,56 | 3,03 |
| 1887,24 | 1639,20 | 91,67 | 11,69 | 16,81 | 73,31 | 32,15 | 66,21 | 3,40 | 30,64 | 2,07 |
| 1574,70 | 1521,12 | 90,81 | 11,75 | 11,90 | 69,92 | 36,63 | 59,67 | 3,48 | 31,05 | 3,00 |
| 790,34 | 1705,28 | 91,44 | 11,92 | 15,32 | 73,00 | 32,71 | 62,70 | 3,32 | 30,28 | 2,55 |
| 1528,60 | 1528,98 | 91,95 | 10,90 | 14,61 | 69,64 | 32,15 | 61,96 | 3,04 | 31,30 | 2,48 |
| 1618,06 | 1618,46 | 90,98 | 10,85 | 14,20 | 74,26 | 34,50 | 57,00 | 3,96 | 33,08 | 2,07 |
| 1410,77 | 1217,79 | 91,04 | 12,37 | 14,20 | 71,27 | 34,94 | 58,95 | 3,49 | 30,82 | 2,60 |
| 1880,78 | 2121,08 | 91,69 | 12,78 | 9,68 | 79,81 | 42,50 | 51,23 | 4,10 | 35,69 | 3,33 |
| 1868,72 | 996,72 | 92,47 | 11,46 | 9,58 | 79,27 | 41,63 | 50,03 | 4,19 | 35,27 | 2,80 |
| 1670,13 | 1312,36 | 94,12 | 9,96 | 8,48 | 78,33 | 39,88 | 55,33 | 3,78 | 34,22 | 1,73 |
| 1759,46 | 2251,74 | 92,86 | 10,52 | 9,35 | 78,16 | 39,01 | 56,88 | 3,78 | 33,97 | 1,83 |
| 1902,83 | 1731,60 | 92,42 | 11,84 | 11,90 | 74,30 | 38,50 | 56,30 | 3,80 | 33,40 | 2,48 |
| 1724,30 | 1370,57 | 92,18 | 12,86 | 12,76 | 74,35 | 37,74 | 58,34 | 3,67 | 33,08 | 2,91 |
| 1939,59 | 1747,73 | 91,93 | 10,96 | 14,06 | 72,41 | 35,04 | 60,06 | 3,36 | 31,38 | 1,78 |
| 1595,55 | 1193,02 | 92,73 | 11,26 | 10,80 | 73,97 | 39,95 | 57,60 | 3,73 | 33,65 | 2,40 |
| 1183,10 | 944,69 | 91,05 | 12,04 | 9,11 | 75,77 | 43,47 | 51,00 | 4,34 | 36,19 | 3,93 |
| 1488,81 | 1156,85 | 91,29 | 10,83 | 12,59 | 77,67 | 39,87 | 51,88 | 4,49 | 35,08 | 2,36 |
| 2148,47 | 1445,94 | 93,58 | 11,55 | 12,29 | 74,01 | 36,80 | 55,63 | 3,86 | 32,76 | 2,05 |
| 1817,91 | 1569,44 | 92,29 | 10,99 | 11,72 | 72,26 | 36,38 | 59,34 | 3,47 | 31,59 | 2,11 |
| 1170,67 | 1201,77 | 91,43 | 12,94 | 16,69 | 67,65 | 34,80 | 70,48 | 3,09 | 30,50 | 3,14 |
| 1113,98 | 1143,57 | 92,02 | 12,34 | 13,37 | 74,46 | 37,23 | 60,44 | 3,63 | 33,34 | 2,76 |
| 1479,41 | 1009,61 | 91,83 | 11,57 | 12,81 | 74,86 | 37,92 | 57,25 | 3,81 | 33,25 | 2,60 |
| 1514,01 | 1243,51 | 91,73 | 12,14 | 13,40 | 72,20 | 36,44 | 64,02 | 3,25 | 31,71 | 2,78 |
| 1313,42 | 1326,40 | 92,41 | 12,04 | 14,00 | 74,09 | 36,70 | 60,58 | 3,60 | 32,33 | 2,75 |
| 1041,78 | 1052,08 | 92,21 | 11,21 | 14,18 | 73,90 | 37,98 | 56,08 | 4,11 | 33,70 | 2,23 |
| 1675,36 | 876,48 | 91,72 | 11,83 | 12,72 | 71,83 | 37,37 | 60,11 | 3,54 | 31,75 | 2,65 |
| 1828,24 | 928,05 | 90,24 | 12,37 | 11,27 | 73,90 | 37,10 | 50,26 | 3,70 | 35,97 | 3,90 |
| 1720,19 | 954,07 | 91,28 | 11,16 | 12,18 | 73,90 | 38,93 | 51,95 | 3,70 | 34,63 | 2,71 |

| | | | | | | | | | | |
|---|---|---|---|---|---|---|---|---|---|---|
| 1484,31 | 823,25 | 91,28 | 11,16 | 13,22 | 75,96 | 37,76 | 56,91 | 3,92 | 34,49 | 2,15 |
| 2320,64 | 1006,29 | 91,07 | 11,67 | 15,23 | 75,66 | 35,97 | 55,53 | 3,97 | 33,20 | 2,54 |
| 1655,51 | 1208,79 | 92,37 | 11,15 | 11,07 | 73,02 | 36,89 | 58,98 | 3,64 | 32,47 | 2,05 |
| 1712,36 | 1193,43 | 90,72 | 12,90 | 14,70 | 69,19 | 36,40 | 62,18 | 3,36 | 31,36 | 3,33 |
| 1732,58 | 1443,71 | 91,59 | 12,06 | 14,03 | 74,18 | 36,75 | 62,32 | 3,45 | 32,19 | 2,88 |
| 1741,32 | 944,76 | 91,89 | 12,87 | 14,70 | 68,33 | 32,76 | 64,23 | 3,05 | 29,10 | 3,22 |
| 1566,67 | 688,75 | 92,86 | 13,15 | 15,54 | 70,36 | 36,00 | 68,03 | 3,24 | 31,62 | 2,86 |
| 1580,62 | 842,55 | 90,97 | 11,72 | 14,72 | 72,28 | 37,26 | 65,27 | 3,68 | 32,71 | 2,04 |
| 1842,50 | 1693,80 | 92,41 | 11,23 | 12,95 | 75,58 | 38,34 | 55,98 | 4,08 | 33,58 | 2,16 |
| 1425,19 | 1310,17 | 91,60 | 11,84 | 14,16 | 72,62 | 36,50 | 64,25 | 3,39 | 32,19 | 2,09 |
| 1441,35 | 812,29 | 91,73 | 10,98 | 13,40 | 75,07 | 37,51 | 58,29 | 3,73 | 33,08 | 1,79 |
| 1227,34 | 831,03 | 90,10 | 11,56 | 15,92 | 72,80 | 36,51 | 56,50 | 4,03 | 33,15 | 3,20 |
| 1811,26 | 1663,53 | 90,74 | 10,95 | 15,03 | 72,71 | 35,28 | 59,25 | 3,49 | 31,46 | 2,63 |
| 1559,49 | 1432,30 | 91,58 | 11,11 | 15,59 | 72,10 | 34,10 | 61,07 | 3,51 | 31,21 | 2,41 |
| 1526,26 | 951,00 | 91,51 | 11,27 | 14,70 | 75,33 | 36,20 | 53,34 | 3,60 | 34,62 | 2,64 |
| 1947,47 | 1213,45 | 91,35 | 12,54 | 15,75 | 73,26 | 37,24 | 60,39 | 3,68 | 32,21 | 3,22 |
| 1901,10 | 1184,56 | 92,32 | 12,10 | 16,55 | 69,98 | 35,20 | 61,85 | 3,69 | 31,03 | 2,40 |
| 2415,05 | 1504,79 | 91,55 | 11,54 | 13,82 | 75,78 | 37,65 | 54,28 | 3,89 | 33,86 | 2,78 |
| 2070,93 | 1625,06 | 91,60 | 11,35 | 13,06 | 74,63 | 34,92 | 57,66 | 3,28 | 32,22 | 2,08 |
| 1988,71 | 1028,01 | 93,49 | 11,39 | 15,28 | 72,54 | 31,12 | 66,48 | 2,65 | 29,65 | 1,74 |
| 2041,03 | 1841,60 | 92,72 | 10,93 | 15,43 | 74,38 | 32,63 | 61,15 | 3,55 | 30,97 | 1,73 |
| 1519,64 | 1767,66 | 92,31 | 11,19 | 12,50 | 71,08 | 36,42 | 60,28 | 3,52 | 31,07 | 2,08 |
| 1661,47 | 1447,55 | 92,17 | 12,40 | 13,37 | 69,92 | 36,73 | 57,90 | 3,51 | 30,99 | 3,33 |
| 1689,48 | 1965,22 | 91,18 | 11,09 | 12,90 | 70,50 | 39,29 | 52,57 | 3,66 | 32,79 | 3,21 |
| 1537,27 | 839,83 | 92,67 | 11,24 | 14,80 | 72,50 | 36,94 | 55,74 | 3,80 | 32,75 | 2,49 |
| 1436,45 | 1616,90 | 92,01 | 11,75 | 13,93 | 72,66 | 36,31 | 58,62 | 3,39 | 32,29 | 3,02 |
| 1472,51 | 1657,49 | 91,97 | 13,57 | 15,59 | 72,33 | 36,26 | 58,12 | 3,56 | 31,54 | 3,98 |
